# The effect of iron-fortified complementary food and intermittent preventive treatment of malaria on anaemia in 12- to 36-month-old children: a cluster-randomised controlled trial

**DOI:** 10.1186/s12936-015-0872-3

**Published:** 2015-09-17

**Authors:** Dominik Glinz, Richard F. Hurrell, Mamadou Ouattara, Michael B. Zimmermann, Gary M. Brittenham, Lukas G. Adiossan, Aurélie A. Righetti, Burkhardt Seifert, Victorine G. Diakité, Jürg Utzinger, Eliézer K. N’Goran, Rita Wegmüller

**Affiliations:** Human Nutrition Laboratory, Institute of Food, Nutrition and Health, ETH Zurich, Schmelzbergstrasse 7, 8092 Zurich, Switzerland; Unité de Formation et de Recherche Biosciences, Université Félix Houphouët-Boigny, Abidjan, Côte d’Ivoire; Department of Pediatrics, Columbia University College of Physicians and Surgeons, New York, USA; Hôpital Général de Taabo, Taabo Cité, Côte d’Ivoire; Department of Epidemiology and Public Health, Swiss Tropical and Public Health Institute, Basel, Switzerland; University of Basel, Basel, Switzerland; Department of Biostatistics, Epidemiology, Biostatistics and Prevention Institute, University of Zurich, Zurich, Switzerland; Université Alassane Ouattara, Bouaké, Côte d’Ivoire; Centre Suisse de Recherches Scientifiques en Côte d’Ivoire, Abidjan, Côte d’Ivoire

**Keywords:** Anaemia, Complementary food, Côte d’Ivoire, Haemoglobin, Intermittent preventive treatment, Iron deficiency, Iron fortification, Malaria, Plasma ferritin, *Plasmodium falciparum*

## Abstract

**Background:**

Iron deficiency (ID) and malaria co-exist in tropical regions and both contribute to high rates of anaemia in young children. It is unclear whether iron fortification combined with intermittent preventive treatment (IPT) of malaria would be an efficacious strategy for reducing anaemia in young children.

**Methods:**

A 9-month cluster-randomised, single-blinded, placebo-controlled intervention trial was carried out in children aged 12–36 months in south-central Côte d’Ivoire, an area of intense and perennial malaria transmission. The study groups were: group 1: normal diet and IPT-placebo (n = 125); group 2: consumption of porridge, an iron-fortified complementary food (CF) with optimised composition providing 2 mg iron as NaFeEDTA and 3.8 mg iron as ferrous fumarate 6 days per week (CF-FeFum) and IPT-placebo (n = 126); group 3: IPT of malaria at 3-month intervals, using sulfadoxine-pyrimethamine and amodiaquine and no dietary intervention (n = 127); group 4: both CF-FeFum and IPT (n = 124); and group 5: consumption of porridge, an iron-fortified CF with the composition currently on the Ivorian market providing 2 mg iron as NaFeEDTA and 3.8 mg iron as ferric pyrophosphate 6 days per week (CF-FePP) and IPT-placebo (n = 127). The primary outcome was haemoglobin (Hb) concentration. Linear and logistic regression mixed-effect models were used for the comparison of the five study groups, and a 2 × 2 factorial analysis was used to assess treatment interactions of CF-FeFum and IPT (study groups 1–4).

**Results:**

After 9 months, the Hb concentration increased in all groups to a similar extent with no statistically significant difference between groups. In the 2 × 2 factorial analysis after 9 months, no treatment interaction was found on Hb (*P* = 0.89). The adjusted differences in Hb were 0.24 g/dl (95 % CI −0.10 to 0.59; *P* = 0.16) in children receiving IPT and −0.08 g/dl (95 % CI −0.42 to 0.26; *P* = 0.65) in children receiving CF-FeFum. At baseline, anaemia (Hb <11.0 g/dl) was 82.1 %. After 9 months, IPT decreased the odds of anaemia (odds ratio [OR], 0.46 [95 % CI 0.23–0.91]; *P* = 0.023), whereas iron-fortified CF did not (OR, 0.85 [95 % CI 0.43–1.68]; *P* = 0.68), although ID (plasma ferritin <30 μg/l) was decreased markedly in children receiving iron fortified CF (OR, 0.19 [95 % CI 0.09–0.40]; *P* < 0.001).

**Conclusions:**

IPT alone only modestly decreased anaemia, but neither IPT nor iron fortified CF significantly improved Hb concentration after 9 months. Additionally, IPT did not augment the effect of the iron fortified CF. CF fortified with highly bioavailable iron improved iron status but not Hb concentration, despite three-monthly IPT of malaria. Thus, further research is necessary to develop effective combination strategies to prevent and treat anaemia in malaria endemic regions.

Trial registration: http://www.clinicaltrials.gov; identifier NCT01634945; registered on July 3, 2012.

**Electronic supplementary material:**

The online version of this article (doi:10.1186/s12936-015-0872-3) contains supplementary material, which is available to authorised users.

## Background

Anaemia in sub-Saharan Africa has many aetiologies, but iron deficiency (ID) and malaria are considered to be the major causes [1]. ID has been estimated to be responsible for more than one-third, and *Plasmodium falciparum* malaria for 24 % of all anaemia cases [1]. *P. falciparum* is highly endemic in rural Côte d’Ivoire [2] and is a major risk factor for anaemia in young children [3]. The proportion of anaemia attributed to these two causes varies with setting and population group [3, 4], and anaemia at the individual level may well result from both aetiologies.

Anaemia can be reduced in settings of seasonal transmission by intermittent preventive treatment (IPT) of malaria [5]. Iron fortification may reduce the prevalence of anaemia in children aged below 2 years [6], but its effectiveness in sub-Saharan Africa remains uncertain. Iron stable isotope studies have reported that inflammation during afebrile malaria in women [7] and children [8] increases circulating hepcidin and decreases iron absorption. Therefore, IPT of malaria might improve the response to iron fortification in areas where malaria is highly endemic.

The aim of this study was to assess the effect of a combination of two highly bioavailable iron compounds [9] (i.e. CF-FeFum: ferrous fumarate combined with NaFeEDTA) added to a cereal-based complementary food (CF) and IPT of malaria using sulfadoxine-pyrimethamine and amodiaquine (SP/AQ) administered once every 3 months. The interventions were given alone or in combination. The primary and secondary outcomes were assessed for treatment interaction using a 2 × 2 factorial analysis. The primary outcome measure was haemoglobin (Hb) concentration. Secondary outcomes were iron status markers and *Plasmodium* prevalence. The study was conducted in children aged 12–36 months living in a rural part of south-central Côte d’Ivoire, where malaria transmission is perennial. The hypothesis was that a combined intervention with IPT of malaria and iron fortified CF would reduce anaemia and ID prevalence to a greater extent than either intervention alone. A fifth study group was included to test the efficacy of the combination of NaFeEDTA and ferric pyrophosphate (CF-FePP), a combination that is currently being used on the Ivorian market. Ferric pyrophosphate is considered to have a lower bioavailability than ferrous fumarate [9].

## Methods

### Study design and participants

**Fig. 1:**
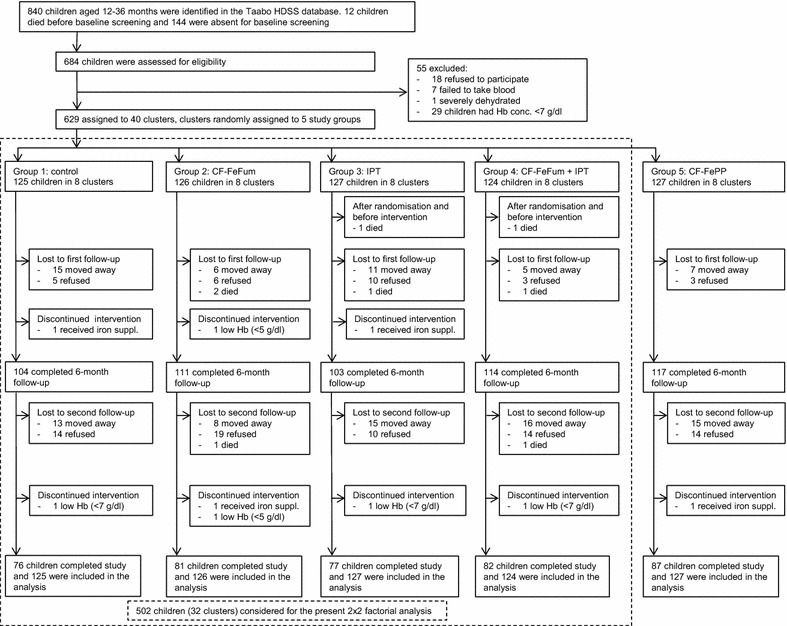
Trial profile. *CF-FeFum* complementary food fortified with NaFeEDTA + ferrous fumarate; *CF-FePP* complementary food fortified with NaFeEDTA + ferric pyrophosphate, *IPT* intermittent preventive treatment of malaria, *Hb* haemoglobin, *HDSS* health and demographic surveillance system

This 9-month cluster-randomised, single-blinded, placebo-controlled intervention trial was conducted in the Taabo health and demographic surveillance system (HDSS) in south-central Côte d’Ivoire [10]. Recently it has been reported that the leading causes of death among children below 5 years of age in the Taabo HDSS are malaria, followed by acute respiratory tract infections and HIV/AIDS [11]. Malaria transmission is perennial with peaks occurring in the large rainy season (from April to August) and in the small rainy season (September/October). The prevalence of *P.* *falciparum* infection ranges between 35 and 77 %, depending on season and age group [12, 13]. From the Taabo HDSS database, we identified 840 children aged 12–36 months in five villages, which were invited for baseline screening (Fig. 1) from mid-April to mid-May 2012. Inclusion criteria were: (1) Hb ≥7 g/dl; (2) no major chronic illnesses; (3) anticipated residence in the area for the entire study duration; and (4) no known allergies to sulfadoxine, pyrimethamine and amodiaquine.

At baseline screening, 629 eligible children were identified and were grouped into 40 clusters based on proximity of their residence, with at least five clusters in each of the five villages and from 13 to 18 children in each cluster. Then the clusters were randomly assigned to five study groups by drawing cluster numbers from an opaque hat (“urn randomisation”) together with village authorities acting as witnesses. This public randomisation process was implemented to avoid any feelings of unfairness among the village population. Allocation ratio was 1:1 for all five study groups. The study groups were: group 1: normal diet (no dietary intervention) + IPT-placebo; group 2: iron fortified CF-FeFum + IPT-placebo; group 3: normal diet + IPT; group 4: CF-FeFum + IPT; and group 5: CF-FePP + IPT-placebo. Interventions lasted for 9 months.

### Ethical considerations

The study was approved by the ethics committees of ETH Zurich (reference no. EK 2009-N-19) and Côte d’Ivoire (reference no. 061 MSLS/CNER). Village and health authorities, and parents/guardians of participating children were informed about the purpose, procedures, and potential risks and benefits of the study. Parental written informed consent was obtained for each child before randomisation. The trial was registered at clinicaltrials.gov (identifier NCT01634945). An independent data safety and monitoring board assessed study progress, provided expertise and assessed the safety of the interventions.

### Complementary food production, preparation and feeding

The roller-dried maize- and soy-flour based CF was produced at Protein Kissèe-La (PKL) in Abidjan. Detailed porridge production procedures and composition are described in additional tables (see Additional file 1). The iron fortified CF contained 2.0 mg iron as NaFeEDTA, 3.8 mg iron as ferrous fumarate, and 0.6 mg native iron in a daily serving of 25 g dry weight porridge. The commercial iron fortified CF-FePP (study group 5) contained 2.0 mg iron as NaFeEDTA, 3.8 mg iron as ferric pyrophosphate, and 0.6 mg native iron in a daily serving of 25 g dry weight porridge (see Additional file 1). The children consumed the porridge once a day in the early morning, 6 days a week providing 38.4 mg fortification iron per week (6.4 mg per serving). The native and fortificant iron dose of 6.4 mg per day covered 110 % of the recommended nutrient intake (RNI) for children aged 1–3 years, assuming an intermediate iron bioavailability of 10 % [14]. Children assigned to study groups 2 and 4 received CF-FeFum. Children assigned to study group 5 received CF-FePP.

In each of the 16 clusters where children (n = 250) received the CF-FeFum and in eight clusters where children (n = 127) received CF-FePP, one cooking area was installed. Trained women cooks prepared servings (25 mg dry matter) for each child individually, supervised the feeding, and recorded the daily amount of uneaten porridge for each child. Staff from Taabo HDSS monitored all cooking locations and cooks daily. The study investigator (or a study team member) monitored the progress of the study at least once every week.

Children allocated to study groups 1 and 3 received no nutritional intervention and the families were instructed to continue their normal dietary habits. From previous studies in Côte d’Ivoire, it has been found that young children (2–5 years) in rural areas consume mainly cassava, plantain and sauces prepared with okra or peanuts [15, 16], and have estimated a mean iron intake of 5.5 mg/day and a mean phytic acid intake of 107 mg/day [15, 16]. This represents about 45–90 % of their recommended daily intake, assuming a dietary iron absorption from 5 to 10 %.

### IPT of malaria

At baseline, 3 and 6 months post-intervention, children assigned to IPT groups (groups 3 and 4) received one dose of SP (500 mg sulfadoxine plus 25 mg pyrimethamine, or half of the dose if body weight ≤10 kg), and three daily doses of amodiaquine (200 mg on days 1 and 2, and 100 mg on day 3, or half of the dose if body weight ≤10 kg). Children assigned to groups 1, 2 and 5 received identical-appearing placebo tablets.

The trial medications (IPT and placebo) were purchased from Kinapharma Limited (Accra, Ghana). They were coded using different colours for blister packs and packaging. To mask the bitter taste of amodiaquine, the trial medication was dissolved in approximately 4 ml Fanta (SOLIBRA; Abidjan, Côte d’Ivoire). The first dose was administered under supervision of a local nurse, the second and third doses were administered by children’s mothers/guardians. The mothers/guardians were asked to keep empty pill packages for verification of drug administration. This was done by staff from Taabo HDSS, who visited all children 2 days after the first administration. Children who vomited or did not complete treatment were re-dosed.

### Blinding of treatments and follow-up

Since half of the children did not receive the CF, this treatment was not blinded to either subjects or investigators. IPT was single blinded, i.e. the subject, staff from the HDSS, any caregivers (except study physician, see below) and the study nurses who administered the drugs were blinded, but the study supervisor (DG) and physician (LGA) had access to study group assignment. The concealment for IPT was assured by using two different colors for packaging (see above “IPT of malaria”). Sick children reporting to the local health centres were treated according to national guidelines irrespective of their study group assignment.

Mothers/guardians of participating children were encouraged to refer the child to the nearest health centre as soon as the child presented a symptom of illness, especially fever, and report the sick visit to Taabo HDSS staff. Each participating child received a study identity card to facilitate subject identification. At the time of study implementation, all consultations and treatments for children aged <5 years were free of charge in Côte d’Ivoire.

### Outcomes and laboratory methods

Biomedical parameters were investigated at baseline, after 6 months, and after 9 months. The follow-up blood samples were taken within a 2-week period, i.e. at 6 months starting end of November until early December 2012, and at 9 months starting at the beginning of March 2013. The specified primary outcome was Hb concentration. Hb concentration, using a COULTER^®^ Ac·T diff2™ (Beckman Coulter Inc.; Brea, USA), was measured. Anaemia was defined by Hb concentration below 11.0 g/dl [17]. Secondary outcomes were iron status marker and *Plasmodium* prevalence.

Thick and thin blood films were stained with Giemsa and were examined microscopically for *Plasmodium* species and parasitaemia, as described elsewhere [18]. Most of the infections were expected to be with *P.* *falciparum* since infections with *Plasmodium* *ovale* and *Plasmodium malariae* are rare [2]. Clinical malaria was defined as a positive rapid diagnostic test (RDT; ICT ML01 malaria Pf kit; ICT Diagnostics, Cape Town, South Africa) and a tympanic temperature >37.5 °C or recent fever episodes reported by the mother/guardian.

The iron status marker plasma ferritin (PF), and the inflammation status markers α-1-acid-glycoprotein (AGP) and C-reactive protein (CRP), were measured with a sandwich enzyme-linked immunosorbent assay (ELISA), as described elsewhere [19]. ID was defined as PF <30 µg/l, according to recommendations by the World Health Organization (WHO) of ID in populations with high prevalence of infectious diseases [20]. Concentrations below the detection limit were reported as detection limit. CRP above 5 mg/l or AGP concentrations above 1 g/l was considered as inflammation.

### Sample size and statistical analysis

The sample size calculation was based on a previous study among infants (aged 6–24 months) in this region of Côte d’Ivoire [12]. In that study, the standard deviation (SD) was 2.0 g/dl at a mean Hb concentration of 9.7 g/dl. The aim was to detect an Hb difference of 0.8 g/dl, so allowing for a dropout rate of 20 %, it was estimated that 125 children per group were needed to achieve a power level of 90 % at a 5 % level of significance.

After baseline assessment, it was decided to randomise the children into clusters for practical reasons and hence, we deviated from the protocol with randomisation on individual level. The effect of clusters was taken into account with multi-level modelling, whereas individuals are first and clusters the second level (as described below).

Data were analysed with STATA version 13.1 (StataCorp LP; College Station, USA), including all randomised children. The residuals of the continuous variables were assessed for normality. Variables with non-normal distributed residuals were logarithmically transformed. An intention-to-treat analysis was pursued with mixed (fixed and random) linear regression multi-level models to account for random effects due to repeated measures within clusters (clusters are second level). A logistic regression was used taking into account random effect for analysis of prevalence (i.e. binary) data. For comparisons between groups, the models include study group assignment as fixed effects and children’s age as covariate. CRP was added as covariate for analysis of PF as outcome variable.

Treatment interaction (CF-FeFum × IPT) was assessed by including study groups 1–4 in a 2 × 2 factorial analysis. Continuous outcomes are presented as adjusted differences and log-transformed outcomes as adjusted ratios. The mixed-effect multi-level models for the 2 × 2 analysis include intervention as fixed effects and children’s age as covariate (clusters are second level). CRP was added as covariate for analysis of PF as outcome variable. All models for the 2 × 2 factorial analysis were assessed for treatment interactions (CF-FeFum × IPT × time) for primary and secondary outcomes. If no interaction was found, the 2 × 2 factorial analyses were presented. In case of interaction, only the group comparison was presented.

## Results

Six hundred eighty-four children were assessed for eligibility, of which 629 children were assigned to 40 clusters; these clusters were randomly assigned to five groups. Baseline characteristics are summarised in Table 1.

**Table 1:** Baseline characteristics of the randomised study groups

Intervention(s)	Group 1 (n = 125)		Group 2 (n = 126)		Group 3 (n = 127)		Group 4 (n = 124)		Group 5 (n = 127)	
	Control		CF-FeFum		IPT		CF-FeFum + IPT		CF-FePP	
Clusters	8		8		8		8		8	
Girls	65	(52.0 %)	57	(45.2 %)	65	(51.2 %)	65	(52.4 %)	64	(50.2 %)
Age (mo)	23.3	(6.9)	23.5	(7.2)	22.9	(7.0)	23.8	(6.4)	23.4	(7.1)
Haemoglobin conc. (g/dl)	9.8	(1.3)	9.9	(1.2)	9.8	(1.1)	9.9	(1.1)	9.6	(1.2)
Anaemia	102	(81.6 %)	101	(80.2 %)	108	(85.0 %)	105	(84.7 %)	110	(86.6 %)
Plasma ferritin (µg/l)	37.7	(18.3–72.4)	36.2	(21.6–66.0)	37.5	(16.9–74.9)	36.7	(18.2–68.4)	53.0	(28.3–115.7)
Iron deficiency	47	(37.4 %)	50	(40.0 %)	53	(42.1 %)	54	(43.9 %)	34	(26.8 %)
*Plasmodium* prevalence	78	(62.1 %)	73	(57.7 %)	78	(61.4 %)	66	(53.3 %)	84	(66.1 %)
*P. falciparum* parasitaemia^a^	1200	(208–5200)	600	(128–3400)	2240	(880–6920)	688	(240–4000)	2140	(400–7500)
Height (cm)	79.2	(6.8)	78.5	(7.5)	79.7	(6.7)	79.7	(6.6)	78.6	(6.8)
Body weight (kg)	10.5	(9.0–12.0)	11.0	(9.0–12.0)	11.0	(9.5–13.0)	10.8	(9.5–12.4)	10.0	(9.0–12.0)

Data are mean (SD), *n* (%) or median (interquartile range). Anaemia: Hb concentration <11 g/dl; iron deficiency: plasma ferritin <30 µg/l

*CF-FeFum* complementary food fortified with NaFeEDTA + ferrous fumarate, *CF-FePP* complementary food fortified with NaFeEDTA + ferric pyrophosphate, *IPT* intermittent preventive treatment of malaria

^a^parasites/µl of blood, only presented for infected children

Compliance rates of porridge consumption were 92.5, 95.0 and 94.8 % for the study porridge administered to groups 2, 4 and 5, respectively. Compliance with IPT and IPT-placebo for study groups 1–5 were 97.6, 96.0, 96.9, 97.6 and 96.8 % at the first treatment (baseline), 95.2, 94.4, 92.9, 92.0 and 93.7 % at 3 months, and 91.1, 88.1, 81.1, 84.0 and 90.6 % at 6 months. Reasons for non-compliance of IPT of malaria included: children refusing to swallow the drug, vomiting after repeated dosing, or mothers/guardians did not consider the IPT important for their children.

As shown in Fig. 1, about one-third of the children were lost to follow-up over the 9 months intervention. Protocol deviation concerned nine children (1.4 %) who discontinued the interventions: three children were excluded because of Hb <7 g/dl over the course of the study and were treated with iron supplementation; two were excluded because of Hb <5 g/dl and were treated with blood transfusions; and four were excluded because they received iron supplements by the parents/guardians. Eight children died and the cause of death was determined by verbal autopsy. Two deaths occurred before the intervention started but after baseline screening and randomisation. The remaining six deaths occurred over the course of the 9-month study period: none died in groups 1 and 5; in group 2, two died of pneumonia and one from malaria; in group 3, one died from malaria with subsequent anaemia; and in group 4, two died from malaria.

### Haemoglobin and anaemia

**Fig. 2:**
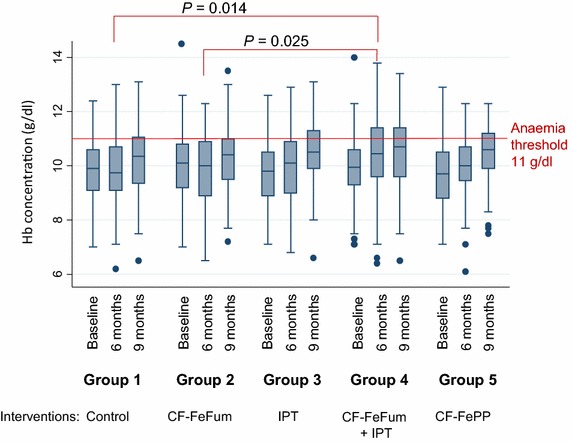
Haemoglobin concentration at baseline, 6 and 9 months for each study group. *CF-FeFum* complementary food fortified with NaFeEDTA + ferrous fumarate, *CF-FePP* complementary food fortified with NaFeEDTA + ferric pyrophosphate, *IPT* intermittent preventive treatment of malaria, *Hb* haemoglobin

Haemoglobin concentration did not differ between the five groups at baseline. The observed increase in the five groups was not different after 9 months (Table 2). After 6 months, a transient effect (during the rainy season) was observed, when Hb concentration increased significantly in children in group 4 (receiving IPT and CF-FeFum) when compared to group 1 (control, *P* = 0.014) or group 2 (children receiving CF-FeFum, *P* = 0.025) (Fig. 2). After 9 months, the decrease of anaemia was significantly higher in groups 3, 4 and 5 than in groups 1 or 2 (Table 2).

**Table 2:** Group comparison analysis

	Group 1	Group 2	Group 3	Group 4	Group 5
	Control	CF-FeFum	IPT	CF-FeFum + IPT	CF-FePP
Participants (n)					
Baseline	125	126	127	124	127
6 months	104	111	103	114	117
9 months	76	81	77	82	87
Hb concentration (g/dl)					
Baseline	9.8 ± 1.3	9.9 ± 1.2	9.8 ± 1.1	9.9 ± 1.1	9.6 ± 1.2
6 months	9.9 ± 1.3	9.9 ± 1.3	10.0 ± 1.3	10.4 ± 1.4*^, Ψ^	10.0 ± 1.1
9 months	10.3 ± 1.3	10.4 ± 1.2	10.5 ± 1.2	10.5 ± 1.2	10.5 ± 1.2
Anaemia					
Baseline	81.6 %	80.2 %	85.0 %	84.7 %	86.6 %
6 months	79.8 %	77.5 %	77.7 %	62.3 %*^, Ψ, †^	81.9 %^¥^
9 months	71.1 %	70.4 %	63.6 %*^, Ψ^	56.1 %**^, ΨΨ^	65.5 %*^, Ψ^
Plasma ferritin (µg/l)					
Baseline	37.7 (18.3–72.4)	36.2 (21.6–66.0)	37.5 (16.9–74.9)	36.7 (18.2–68.4)	53.0 (28.4–115.7)^†, ¥^
6 months	60.7 (35.1–114.0)	102.4 (48.3–159.5)**	56.5 (26.9–92.9)^ΨΨ^	70.9 (42.5–133.0)*^, †^	69.1 (41.8–139.7)^Ψ, ¥^
9 months	49.6 (26.2–96.0)	66.5 (45.4–117.4)	39.4 (23.6–69.3)^ΨΨ^	62.8 (39.2–92.4)^†^	62.6 (41.1–107.2)^¥^
Iron deficiency					
Baseline	37.4 %	40.0 %	42.1 %	43.9 %	26.7 %^†, ¥^
6 months	19.2 %	5.4 %**	27.5 %^ΨΨ^	12.3 %^††^	8.6 %^††^
9 months	29.3 %	3.7 %***	36.4 % ^ΨΨ^	15.8 %^Ψ,††^	10.3 %**^, ††^
Anaemia and iron deficiency					
Baseline	33.3 %	32.8 %	31.7 %	38.2 %	23.6 %^¥^
6 months	15.4 %	3.6 %**	19.6 % ^ΨΨ^	4.4 %**,^†††^	4.3 %*^,††^
9 months	18.7 %	1.2 %**	26.0 % ^ΨΨ^	3.7 %**,^††^	3.4 %**^, ††^
CRP (mg/l)					
Baseline	2.8 (1.0–11.1)	3.4 (1.4–8.7)	4.2 (1.4–12.0)	3.0 (1.1–7.3)	5.9 (1.9–21.3)*^,Ψ,¥^
6 months	5.1 (1.8–18.6)	4.6 (1.2–20.4)	3.6 (1.2–17.3)	3.8 (0.8–17.0)	4.8 (1.5–14.6)*^,Ψ^
9 months	2.6 (1.0–7.3)	4.3 (1.0–13.2)	1.8 (0.5–5.9)	1.8 (0.9–5.5)	3.2 (1.3–14.8)
AGP (g/l)					
Baseline	1.12 (0.90–1.40)	1.27 (1.01–1.54)	1.22 (0.96–1.62)	1.16 (0.92–1.51)	1.26 (0.96–1.65)
6 months	1.13 (0.88–1.41)	1.25 (0.92–1.54)	1.23 (0.80–1.55)	1.04 (0.81–1.44)^†^	1.10 (0.85–1.40)
9 months	1.07 (0.78–1.44)	1.13 (0.85–1.36)	1.10 (0.88–1.35)	1.02 (0.79–1.23)*,^††^	1.06 (0.79–1.28)
Inflammation					
Baseline	65.8 %	76.8 %	76.2 %	73.2 %	76.4 %
6 months	72.1 %	74.8 %	69.6 %	57.0 %^Ψ,††^	65.5 %
9 months	57.3 %	64.2 %	63.6 %	56.1 %^††^	57.5 %
Plasmodium prevalence					
Baseline	62.1 %	57.7 %	61.4 %	53.3 %	66.1 %
6 months	62.5 %	55.0 %	44.7 %*	45.6 %*	64.7 %^¥,†^
9 months	44.7 %	46.9 %	35.1 %^Ψ^	34.1 %^Ψ^	47.1 %
*P. falciparum* parasitaemia (parasites/µl blood)					
Baseline	1200 (208–5200)	600 (128–3400)	2240 (880–6920)	688 (240–4000)	2140 (400–7500)*^,¥^
6 months	4160 (1200–10,480)	1880 (720–6360)	4400 (256–17,080)	1820 (480–11,200)	2720 (640–9040)
9 months	2740 (1080–14,640)	2740 (840–8040)	4520 (440–20,320)	1920 (240–4080)^Ψ, †^	4800 (960–9560)^Ψ^

Data are mean ± SD, % or median (IQR). Changes between baseline to 6 months and baseline to 9 months were compared between groups with random effect models. Anaemia: Hb concentration <11 g/dl; iron deficiency: plasma ferritin <30 µg/l; inflammation: CRP ≥5.0 mg/l or AGP ≥1.0 g/l; and *P.* *falciparum* parasitaemia: parasites/µl blood, only presented for infected children at the specific time points

*CF-FeFum* complementary food fortified with NaFeEDTA + ferrous fumarate, *CF-FePP* complementary food fortified with NaFeEDTA + ferric pyrophosphate, *IPT* intermittent preventive treatment of malaria, *AGP* α-1-acid-glycoprotein, *CRP* C-reactive protein, *Hb* haemoglobin concentration, *IQR* interquartile range

*/**/*** Increase/decrease in groups 2, 3, 4 and 5 significantly different compared to increase/decrease in group 1, *P < 0.05, ** *P* < 0.01, *** *P* < 0.001

^Ψ^ ^/ΨΨ/^ ^ΨΨΨ^Increase/decrease in groups 3, 4 and 5 significantly different compared to group 2, ^Ψ^ *P* < 0.05, ^ΨΨ^ *P* < 0.01, ^ΨΨΨ^ *P* < 0.001

^†^^/††^^/†††^Increase/decrease in groups 4 and 5 significantly different compared to group 3, ^†^ *P* < 0.05, ^††^ *P* < 0.01, ^†††^ *P* < 0.001

^¥^^/¥¥^^/¥¥¥^Increase/decrease in group 5 significantly different compared to group 4, ^¥^ *P* < 0.05, ^¥¥^ *P* < 0.01, ^¥¥¥^ *P* < 0.001

Treatment interactions (CF-FeFum × IPT) were assessed with the 2 × 2 factorial analysis including groups 1–4. No interaction was found between interventions for Hb concentration (*P* = 0.89). After 9 months, no effect of interventions on Hb concentration was found. Similar to the five group comparison, we observed a transient effect after 6 months, when Hb concentration increased significantly in children receiving IPT (n = 251; adjusted mean difference 0.35 g/dl [95 % CI 0.04–0.66; *P* = 0.027]). The increase of Hb concentration after 9 months was no longer significant (0.24 g/dl [95 % CI −0.10 to 0.59; *P* = 0.16]) (Table 3). Children receiving IPT showed lower odds of being anemic after 6 and 9 months (odds ratio [OR], 0.46 [95 % CI 0.24–0.90; *P* = 0.023] and OR, 0.46 [95 % CI 0.23–0.90; *P* = 0.024]) (Table 4). The iron fortified CF-FeFum had no effect on anaemia prevalence after 6 and 9 months (OR, 0.64 [95 % CI 0.33–1.25; *P* = 0.19] and OR, 0.85 [95 % CI 0.43–1.68; *P* = 0.64]).

**Table 3:** Main effects of iron fortified CF-FeFum and IPT of malaria

	Effect of iron fortified complementary food		Effect of intermittent preventive treatment of malaria	
	Received CF-FeFum^a^	n = 250	Received IPT^a^	n = 251
	No CF-FeFum^a^	n = 252	IPT-placebo^a^	n = 251
Adjusted^b^ difference of Hb concentration g/dl (95 % CI)				
6 months	0.11	(−0.20 to 0.42; *P* = 0.49)	0.35	(0.04 to 0.66; *P* = 0.027)
9 months	−0.08	(−0.42 to 0.26; *P* = 0.65)	0.24	(−0.10 to 0.59; *P* = 0.16)
Adjusted^c^ ratios of log-transformed PF concentration (95 % CI)				
6 months	1.33	(1.14 to 1.51; *P* = 0.001)	0.95	(0.76 to 1.13; *P* = 0.58)
9 months	1.36	(1.16 to 1.56; *P* < 0.001)	0.91	(0.70 to 1.11; *P* = 0.37)
Adjusted^d^ ratios of log-transformed CRP concentration				
6 months	1.04	(0.63 to 1.45; *P* = 0.84)	0.77	(0.36 to 1.18; *P* = 0.27)
9 months	1.10	(0.65 to 1.55; *P* = 0.67)	0.53	(0.08 to 0.98; *P* = 0.039)

^a^Assignment at baseline

^b^Adjusted for age, Hb concentration at baseline and factorial design

^c^Adjusted for age, C-reactive protein concentration, PF concentration at baseline and factorial design

^d^Adjusted for age, C-reactive protein concentration at baseline and factorial design

The effects were assessed in 12- to 36-month-old Ivorian children at 6 and 9 months. The estimations are based on a 2 × 2 factorial analysis using a linear regression model taking into account random effects

**Table 4:** Odds ratios for anaemia, iron deficiency, malaria and inflammation prevalence

	Effect of iron fortified complementary food		Effect of intermittent preventive treatment of malaria	
	Received CF-FeFum^a^	n = 250	Received IPT^a^	n = 251
	No CF-FeFum^a^	n = 252	IPT-placebo^a^	n = 251
Odds ratios^b^ of anaemia (Hb concentration <11 g/dl) (95 % CI)				
6 months	0.64	(0.33 to 1.25; *P* = 0.19)	0.46	(0.24 to 0.90; *P* = 0.023)
9 months	0.85	(0.43 to 1.68; *P* = 0.64)	0.46	(0.23 to 0.90; *P* = 0.024)
Odds ratios^c^ of iron deficiency (plasma ferritin concentration <30 µg/l) (95 % CI)				
6 months	0.28	(0.14 to 0.56; *P* < 0.001)	1.52	(0.76 to 3.04; *P* = 0.23)
9 months	0.19	(0.09 to 0.40; *P* < 0.001)	1.64	(0.79 to 3.42; *P* = 0.19)
Odds ratios^b^ of malaria prevalence (*P. falciparum*) (95 % CI)				
6 months	1.17	(0.68 to 2.01; *P* = 0.58)	0.59	(0.34 to 1.02; *P* = 0.057)
9 months	1.30	(0.71 to 2.39; *P* = 0.39)	0.61	(0.33 to 1.12; *P* = 0.11)
Odds ratios^b^ of the inflammation status^d^ (95 % CI)				
6 months	0.66	(0.37 to 1.19; *P* = 0.17)	0.52	(0.29 to 0.94; *P* = 0.030)
9 months	0.82	(0.44 to 1.51; *P* = 0.52)	0.77	(0.42 to 1.43; *P* = 0.41)

^a^Assignment at baseline

^b^Adjusted for age

^c^Adjusted for age and CRP concentration

^d^Inflammation is defined as CRP >5 mg/l and/or AGP >1 g/l

Odds ratios were assessed in 12- to 36-month-old Ivorian children at 6 and 9 months. The estimations are based on a 2 × 2 factorial analysis using a logistic regression model taking into account random effects

### Iron status

At baseline, the PF concentration was slightly higher in children in group 5 (receiving CF-FePP) than in children in groups 3 and 4 (Table 2). At 6 months, the PF concentration increased significantly more in groups 2 and 4 than in groups 1, 3 and 5. After 9 months, the increases in groups 2 and 4 were higher than in group 3. The increase after 9 months in group 4 was higher than in group 5 (Table 2). ID decreased significantly in all three groups receiving iron for 9 months (groups 2, 4 and 5). The decrease of ID in group 2 was higher than in group 4 (Table 2).

In the 2 × 2 factorial analysis, no treatment interaction on PF concentration (*P* = 0.55) was found. From baseline to 6 and 9 months, the PF concentration was increased by 33 % (95 % CI 14–51 %) and 36 % (95 % CI 16–56 %), respectively, in children receiving CF-FeFum (Table 3). Children receiving CF-FeFum were significantly less likely to be iron deficient after 6 months (OR, 0.28 [95 % CI 0.14–0.56; *P* < 0.001]) and 9 months (OR, 0.19 [95 % CI 0.09–0.40; *P* < 0.001]) (Table 4). The 2 × 2 factorial analysis showed no effect by IPT on ferritin concentration and ID prevalence.

### *Plasmodium* prevalence and inflammation

*Plasmodium* prevalence was not different between the five study groups at baseline. After 6 months (end of rainy season), the decrease of malaria prevalence in groups 3 and 4 (all receiving IPT) was significantly higher than in groups 1, 2 and 5 (all receiving IPT-placebo) (Table 2). After 9 months, the *Plasmodium* prevalence decreased in all groups, but the decrease in study 2 was lower than in groups 3 and 4. Inflammation parameters (AGP and CRP) in all five groups showed no major differences at baseline, except CRP in group 5 was significantly higher than in groups 1, 2 and 4.

In the 2 × 2 factorial analysis, no interaction between the two treatments on malaria prevalence was found (*P* = 0.78), on AGP concentration (*P* = 0.18) or on CRP (*P* = 0.52). In the 2 × 2 factorial analysis, neither intervention significantly affected the *P.* *falciparum* prevalence at 6 or 9 months follow-up. CRP concentration was reduced by 47 % after 9 months (95 % CI 2–92 %) in children receiving IPT (Table 3). Children receiving IPT had lower odds to have an elevated inflammation status after 6 months (OR, 0.52 [95 % CI 0.29–0.94; *P* = 0.030]), but this effect was not observed after 9 months (Table 4).

**Fig. 3:**
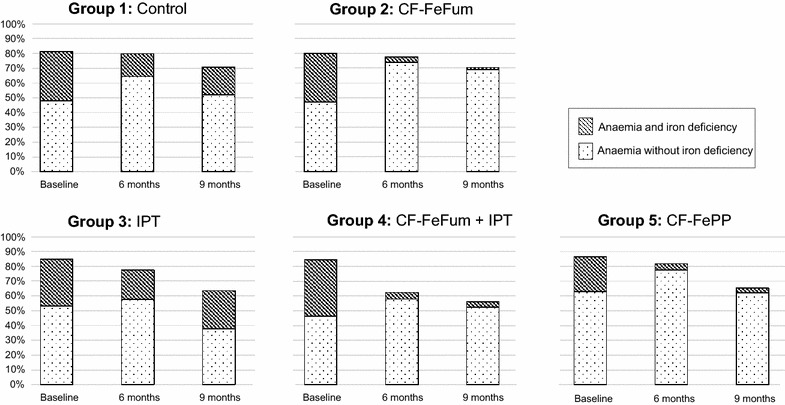
Prevalence of anaemia at baseline, 6 and 9 months for each study group. Anaemic children are separated into children with iron deficiency and no iron deficiency. Iron deficiency was reduced in anaemic children receiving CF-FeFum or CF-FePP. IPT modestly reduced anaemia, without affecting iron deficiency. *CF-FeFum* complementary food fortified with NaFeEDTA + ferrous fumarate, *CF-FePP* complementary food fortified with NaFeEDTA + ferric pyrophosphate, *IPT* intermittent preventive treatment of malaria

Figure 3 shows the prevalence of anaemia in the five study groups. Anaemia was stratified into anaemia with ID (spotted) or anaemia without ID (shaded). This figure displays the dynamic of anaemia with and without ID in the present study population over the 9 months period.

## Discussion

This randomised controlled trial examined the effects of CF fortified with highly bioavailable iron compounds and IPT of malaria in 12- to 36-month-old children in an area of intense malaria transmission in West Africa. To the authors’ knowledge, this is the first trial assessing the treatment interaction of iron-fortified CF and IPT of malaria with a 2 × 2 factorial design. The main findings are: (1) no interaction between IPT of malaria and CF on Hb concentration, anaemia or ID; (2) iron fortified CF had no effect on Hb concentration or anaemia prevalence but substantially decreased the prevalence of ID; (3) IPT of malaria modestly reduced anaemia; and (4) iron fortification with FeFum or FePP had the same effect on Hb concentration, although anaemia decreased more in children receiving FePP.

Despite covering >90 % of the iron RNI with compounds expected to have high bioavailability, after 9 months, iron fortification had no significant effect on Hb concentration or anaemia prevalence. The results differ from a subgroup analysis from a recently published Cochrane review that reported a positive effect of iron fortification at point of consumption with micronutrient powders on anaemia prevalence in malaria-endemic regions [6]. However, micronutrient powders contain more iron than fortified complementary foods and, in the four selected studies utilised for the subgroup analysis, the malaria prevalence was below 10 % in one study, malaria parasitaemia was poorly defined in the other three studies and concomitant anti-malarial treatments were not reported. Hence, the effect of iron fortification on anaemia in malaria-endemic regions remains unclear.

ID was defined as PF <30 µg/l and was based on recommendations from WHO [17] for populations with high prevalence of infections. The prevalence of inflammation in the study population was high; more than two-thirds of the children had an elevated CRP and/or AGP. The potential confounding effect of inflammation was taken into account by adjusting the linear regression models for CRP concentration. Both iron fortified CF increased PF and sharply decreased ID, consistent with two previous studies conducted in Côte d’Ivoire that failed to show a significant effect of iron fortification on Hb concentration in school-aged children but did decrease ID [21, 22].

Treatment with SP/AQ clears *P.* *falciparum* parasitaemia [23], and is protective against reinfection for 28–35 days [24, 25]. Malaria transmission is perennial in this part of Côte d’Ivoire, with high entomological inoculation rates (117–409 infectious bites per person per year) [26]. Hence after the protective effect of the IPT had faded, children were at high risk of re-infection and many of them indeed became re-infected between the IPT treatments. However, the protection of each IPT course was sufficiently long to reduce the risk of anaemia. This protective effect of IPT against anaemia is consistent with a meta-analysis of six IPT trials conducted in areas where malaria transmission is seasonal or perennial with the reported relative risk of anaemia decreased by 21 % (95 % CI 8–33 %) [5]. In the present study, although children assigned to IPT were less likely to be anemic, the increase of the adjusted Hb concentration was not significantly different when compared to children receiving placebo. In other words, at study end, the differences of Hb concentrations were small despite the apparent fall in frequency of anaemia in children receiving IPT (Fig. 2).

No evidence was found that IPT of malaria improves the efficacy of iron fortified CF by facilitating iron absorption or erythropoiesis, which are reported to be diminished during *P.* *falciparum* infection [7, 27]. However, both iron fortified CFs (CF-FeFum and CF-FePP) improved iron status in children similarly regardless of whether or not they received IPT. This finding may be explained by a recent study reporting that serum hepcidin in iron deficient children is decreased, even in the presence of *P.* *falciparum* infection [28]. The lack of an interaction between both interventions on anaemia prevalence is consistent with a previous study in 12- to 16-week-old Tanzanian infants who received iron supplementation with or without IPT of malaria [29]. This study found no additional effect on anaemia when iron supplementation was combined with IPT. Compared to the present study, the Tanzanian children were much younger and iron was given as higher dose supplements over a shorter period. However, the fact that IPT of malaria in the Tanzanian and the current study had a stronger effect on anaemia prevalence than iron alone suggests that infection with *P.* *falciparum* is the predominant risk factor for anaemia in young children.

The present study was implemented at the transition zone of tropical rainforest in the South and the savannah in the North [30] and, although the transmission of malaria is perennial in this part of Côte d’Ivoire, there is also some natural fluctuation in malaria transmission between the dry and the rainy season [3, 31]. The study included the rainy season (baseline to 6 months) and the dry season (6–9 months). The prevalence of *P.* *falciparum* infection in the control group did not change during the first 6 months, but it decreased by almost one-third over the last 3 months. Although Hb concentration did not change in the control group during the first 6 months, it increased during the last 3 months of intervention. The effect of IPT on Hb appeared to be stronger at the end of the rainy season (at 6 months). This suggests the abatement of malaria transmission during the dry season likely influenced the impact of the treatments, and may have been responsible for the differences in outcomes at 6 and 9 months.

The present study has several strengths. It was conducted in infants and young children in a rural setting of West Africa where anaemia is common and malaria transmission is intense. Beside the comparison of the five groups, a 2 × 2 factorial design was employed to assess potential interactions between CF and IPT and compliance was high for both interventions. Iron compounds with high bioavailability were used that had previously demonstrated efficacy [9]. An effective anti-malarial regimen with a relatively long half-life was used, but, nevertheless, many children became re-infected by the time of the next follow-up.

Limitations of the present study include possible confounding by seasonal variations in malaria transmission, the relatively high drop-out rate (attrition bias), and the use of a single iron biomarker (i.e. PF) to define iron status. Inherited haemoglobinopathies are unlikely to play a major role in anaemia in this population, as previous studies in this region reported 83 % of subjects to have normal Hb genotype, and only 8, 7 and 1 % carried an S allele, C allele, and had sickle cell anaemia, respectively [12]. Likewise, other micronutrient deficiencies, such as vitamin A, although present, were not associated with anaemia in the same region [12]. Hookworm contributes to anaemia in many tropical regions, but the prevalence of hookworm was very low in our study subjects, similar to an earlier investigation [12].

## Conclusions

The results of the present study confirm that anaemia is a major public health problem in young children in Côte d’Ivoire and requires urgent action. About half of the anaemic children were iron deficient and the iron fortified CF almost eliminated ID, but against the authors’ expectation, it did not decrease anaemia prevalence. This observation is in line with a recently published Cochrane review including anemic children living in malaria-endemic regions receiving iron supplementation and IPT for a 12-week period [32]. The authors reported a modest effect of IPT on Hb concentration, irrespective of whether the children received supplemental iron (sub-group analysis). The Cochrane review and the current findings argue that the effect of IPT on anaemia should be further investigated, maybe in combination with the reduction of malaria transmission, to be achieved through a combination of vector control strategies (e.g. removal of potential breeding sites, use of long-lasting insecticidal nets, indoor residual spraying and IPT of malaria). The findings of the present study further show that iron fortification of CF is an effective fortification strategy to prevent negative health consequences due to ID. Moreover, these findings underline the multiple aetiologies of anaemia in tropical settings and point to combined interventions being necessary for its prevention or treatment.

## Additional file

Additional file 1. Composition of the dried complementary food and vitamin/mineral premix

## Abbreviations

AGP: α-1-acid-glycoprotein
CF: complementary food
CF-FeFum: complementary food fortified with NaFeEDTA + ferrous fumarate
CF-FePP: complementary food fortified with NaFeEDTA + ferric pyrophosphate
CRP: C-reactive protein
Hb: haemoglobin
ID: iron deficiency
IPT: intermittent preventive treatment of malaria
PF: plasma ferritin
SP/AQ: sulfadoxine-pyrimethamine and amodiaquine
