# Supplementary material for: The effect of iron-fortified complementary food and intermittent preventive treatment of malaria on anaemia in 12- to 36-month-old children: a cluster-randomised controlled trial
Source: Malar J. 2015 Sep 17;14:347. doi: 10.1186/s12936-015-0872-3 (PMC4573684; doi:10.1186/s12936-015-0872-3)
Supplement: Additional file 1. — Composition of the dried complementary food and vitamin/mineral premix [file 12936_2015_872_MOESM1_ESM.docx]

Supplementary appendix

**Additional table 1 – Contents of the dried complementary food**

| **Content** | **Percentage** |
| --- | --- |
| Maize flour | 49.9 |
| Soy flour | 21.4 |
| Sucrose | 20.0 |
| Milk powder | 7.0 |
| Aroma | 0.3 |
| Salt | 0.1 |
| Vitamin/mineral premix | 1.3 |

**Additional table 2 – Mineral and vitamin composition of the fortified complementary food** (the amounts are represented as elemental content)

| **Micronutrient** | **Chemical form** | **Unit** | **Content/25g serving** |
| --- | --- | --- | --- |
| Iron | Ferrous Fumarate | mg | 3.8 |
|  | Sodium Iron EDTA | mg | 2.0 |
|  | Native iron | mg | 0.6 |
| Vitamin A | Vitamin A acetate | IU | 667 |
| Vitamin B1 | Thiamin HCL | mg | 0.25 |
| Vitamin B2 | Riboflavin | mg | 0.25 |
| Vitamin B6 | Pyridoxine HCL | mg | 0.25 |
| Vitamin B12 | Cyanocobalamin (diluted form (0·1% or 1%) | µg | 0.45 |
| Vitamin C | Sodium Ascorbate | mg | 15 |
| Vitamin D | Vitamin D3 | IU | 100 |
| Vitamin E | Vitamin E acetate | mg | 2.5 |
| Niacin | Niacin (niacinamide) | mg | 3 |
| Zinc | Zinc sulfate (1 H_2_O) | mg | 4.15 |
| Iodine | potassium iodide 10% dilution | µg | 45 |
| Folic acid | Folic Acid | µg | 75 |
| Magnesium | Magnesium phosphate dibasic | mg | 30 |
| Copper | Copper sulfate | mg | 0.28 |
| Calcium |  | mg | 66.5 |
| Biotin |  | µg | 4 |
| Pantothenic acid |  | mg | 1 |
| Manganese |  | mg | 0.6 |
|  |  |  |  |

**Mineral and vitamin composition of the fortified complementary food for the 5^th^** study group (the amounts are represented as elemental content)

| **Micronutrient** | **Chemical form** | **Unit** | **Content/25g serving** |
| --- | --- | --- | --- |
| Iron | Micronized ferric pyrophosphate | mg | 3.8 |
|  | Sodium Iron EDTA | mg | 2.0 |
|  | Native iron | mg | 0.6 |
| Vitamin A | Vitamin A acetate | IU | 667 |
| Vitamin B1 | Thiamin HCL | mg | 0.25 |
| Vitamin B2 | Riboflavin | mg | 0.25 |
| Vitamin B6 | Pyridoxine HCL | mg | 0.25 |
| Vitamin B12 | Cyanocobalamin (diluted form (0·1% or 1%) | µg | 0.45 |
| Vitamin C | Sodium Ascorbate | mg | 15 |
| Vitamin D | Vitamin D3 | IU | 100 |
| Vitamin E | Vitamin E acetate | mg | 2.5 |
| Niacin | Niacin (niacinamide) | mg | 3 |
| Zinc | Zinc sulfate (1 H_2_O) | mg | 4.15 |
| Iodine | potassium iodide 10% dilution | µg | 45 |
| Folic acid | Folic Acid | µg | 75 |
| Magnesium | Magnesium phosphate dibasic | mg | 30 |
| Copper | Copper sulfate | mg | 0.28 |
| Calcium |  | mg | 66.5 |
| Biotin |  | µg | 4 |
| Pantothenic acid |  | mg | 1 |
| Manganese |  | mg | 0.6 |
